# The Preservation of the Teeth

**Published:** 1913-07-15

**Authors:** Norman Roberts


### THE PRESERVATION OF THE TEETH.

NORMAN ROBERTS, A.B., M.D.

For several years past the dental profession and certain commercial interests have been waging an admirable publicity campaign on the desirability of constant cleanliness and systematic early repair of the teeth. (If the medical profession has done as well in proportion to its opportunities, we should to-day deservedly stand for higher than we do in the public estimation).This toothbrush crusade is indeed very opportune; but there is something far better along the same line, of which to-day we hear scarcely a whisper, least of all from the dentists, who of late years, by reason of intensive but narrow special training have been allowed to assume a position of authority in a field which still rightly comes under the more comprehensive domain of the physician.

*Clean* teeth are of course not so *apt* to decay as dirty ones; but *strong* teeth can hardly be *made* to decay—a strong statement, but amply justified. To prevent decay by cleanliness alone, the mouth would have to be kept constantly aseptic, a practical impossibility; but strength can be secured with certainty if systematically worked for from childhood up. The obvious way to secure strength is by physiologic use, i. e., by chewing something with adequate resistance and with sufficient taste to stimulate the impulse to chew. The enormous consumption of gum shows the instinctively-felt need of civilized humanity for *more chewing*, but gum lacks resistance; what is needed is something like a fibrous root containing, and when chewed slowly, yielding a nourishing and agreeably-tasting salivary extract, preferably containing much carbohydrate (for the reason that carbohydrate more effectively than anything else physiologically stimulates the flow of the saliva and the impulse to prolonged mastication). The structure of the human teeth, and the known manner of life of many primitive peoples show that human teeth were evolved mainly for just such use; hence they are best to be kept in condition by such use, rather than (though not at all to the exclusion of) such artificial measures as oral asepsis. Moreover, such natural use of the mouth (with the resulting long-continued flow of alkaline saliva) is far more efficient in reducing the numbers and virulence of tooth-destroying bacteria than any *practical* system of artificial oral asepsis; and the slight but sufficient individual motion of the teeth during vigorous and prolonged mastication is in the long run far more effective and less damaging than silk or tooth pick in dislodging fermenting material from between the teeth—although, of course, artificial aids are not to be despised when especially indicated.

A familiar substance that meets the requirements of proper consistency and normal physiological stimulation is *licorice root.* A set of fairly strong teeth can safely break off the sticks into the proper lengths (about an inch) and soon reduce them to a fibrous coherent pulp, which on continued chewing yields the carbohydrate *glycyrrhizin* and other agreeably-flavored extractives during ten or twenty minutes' moderate chewing. If the teeth are too weak to break up the stick, it may be taken in a safer "fine-cut" (*not ground*) condition, which may be chewed as vigorously or as gingerly as desired. Besides, licorice, there are doubtless many other suitable substances already known; and if there were a demand, much better ones could be provided.

The best way to use licorice for this purpose seems to be to chew a comfortable mouthful (swallowing the saliva, of course) until the sweet taste is replaced by slight astringency and the pulp begins to disintegrate and irritate the pharynx. One or two helpings after each meal is sufficient; but no harm is known to result from keeping it up for several hours a day, although of course the salivary glands may be depleted more or less if there is no intermission before meals. The habit may with great advantage be substituted for the use of tobacco; it is beneficial instead of harmful; it gives one "something to do while doing nothing"; it does not interfere with most kinds of work; and it molests no one except a few selfish and supersensitive souls who are irritated at the sight of others quietly enjoying themselves. The main drawback to its extended use by large numbers of people seems to be that it is largely controlled by a soulless trust, who mix a little good root with a great deal of bad, and who would probably boost the price out of sight if the demand were much increased.

There are obvious "medical" as well as "dental" grounds for advocating such a partial return to primitive habits of eating. Modern soft, bland foods do not stimulate the act of mastication or the secretion of saliva enough to cause the carbohydrates to be adequately digested in the stomach during the half hour or so that elapses before the hydrochloric acid becomes so concentrated as to kill the ptyalin, and digestion of carbohydrate is hence delayed until the amylopsin of the pancreatic juice reaches it in the intestines. This delay often results in fermentation and heartburn, which (in at least my own case) is quickly relieved by chewing the licorice. The salivary glands, even in their present neglected condition, secrete much more of the starch-splitting ferment than does the pancreas, and should for every reason be given opportunity to contribute it at the proper time. Of course, it would be better if the carbohydrate ration could all be taken in such form as to require prolonged chewing; and some day such will be the universal custom; but until then it will materially help to take even a small part of it in the manner described.

Certain Sunday paper evolutionists are prone to amuse themselves and turn an honest penny by prophesying the future physical condition of the race when civilization and evolution shall have made us a finished product. We shall be hairless; our limbs will be delicate tentacles of use only to manipulate electric organ keys controlling the machinery that is to obviate all need for gross physical exertion; our sense organs will be superseded by infinitely more delicate electric receiving apparatus, and will atrophy for want of use; our women will simply ovulate as the frogs do, the product of conception being cared for in the womb of some lower animal (or in another of the omnipotent machine) until maturity; and we will live somewhat as the amoeba does, by absorption through our delicate skins of predigested nourishment from a bath, our digestive apparatus having faded entirely away, especially the teeth, against which these great scientists seem to be as violently prejudiced as they are against the hair. Now this tapeworm-like existence may seem highly to be desired by a few people with tapeworm-like minds, but it does *not* appeal to the undersigned or any other normal human being; and as Man will undoubtedly soon completely control evolution throughout the world, it is a foregone conclusion that he will take care of himself in a way to recover the organs and faculties that he has lost, rather than submit himself to further mutilation. Undoubtedly "superfluous" parts and powers make a human being a less tractable and less highly-specialized producer of wealth for his "betters" (hence please note the rising volume of protest from the *influential* against the higher education of the masses, and the substitution of vocational training in the schools); but producing wealth for one's betters is rapidly losing popularity. Soon the practical application of correct economic principles (now opposed by the shortsighted selfish) will convert Machinery and System from cruel tyrants over the bulk of humanity to efficient servants of the whole race. Once before, in Ancient Greece, an entire people was free, though they lived on the labor of alien slaves, and all free citizens delighted to house their splendid minds in perfect bodies. Even more so in the golden future, when all living things shall be free and only machines shall slave; man's animal body will be as perfect as his godlike mind—not a flabby, helpless parasite, to be swept out of existence by the first accident to the sheltering machines, but a real king of the earth, the physical as well as the mental superior of any other live thing on it.

And so we shall keep all our parts and powers, including our teeth; and this rather by intelligent use of them than by coddling and tinkering, although for the next few years or maybe generations the coddling and tinkering may have to be kept up (decreasingly) to atone for the follies of the past. We shall recover all that we need of what we have lost; we shall not need teeth for weapons, nor even for tools, hence we shall be as far removed from projecting tushes and prognathous jaws as from chinlessness and the likeness of spoon-fed senility.

The right time to begin to recover the lost ground is at once. Five cents' worth of licorice root will start any one person (or two or three) on the right track, if he has any serviceable teeth left. Young children should especially be gotten into the practice, as it will not only conserve their already-erupted teeth, but will favor the proper eruption of the later ones, normally develop the whole lower part of the head (bony, muscular and glandular), and tend to break up thumbsucking and mouthbreathing and prevent the development of adenoids and enlarged tonsils.

Is it too much to hope that someone with time and influence now less profitably employed will take this matter up and give it the *initial impetus* that it deserves? *American Medicine.*

